# Associations of Objectively Measured Physical Activity and Sleep with Weight Loss Maintenance: A Preliminary Study of Japanese Adults

**DOI:** 10.3390/bs10010003

**Published:** 2019-12-18

**Authors:** Kyohsuke Wakaba, Hiroyuki Sasai, Yoshio Nakata

**Affiliations:** 1 Faculty of Human Life, Jumonji University, Niiza, Saitama 352-8510, Japan; k-wakaba@jumonji-u.ac.jp; 2 Department of Life Sciences, Graduate School of Arts and Sciences, The University of Tokyo, Meguro, Tokyo 153-8902, Japan; 3 Research Team for Promoting Independence and Mental Health, Tokyo Metropolitan Institute of Gerontology, Itabashi, Tokyo 173-0015, Japan; 4 Faculty of Health and Sport Sciences, University of Tsukuba, Tsukuba, Ibaraki 305-8577, Japan; nakata.yoshio.gn@u.tsukuba.ac.jp

**Keywords:** sleep, sedentary behavior, moderate-to-vigorous physical activity, weight loss maintenance

## Abstract

Maintaining weight loss is considerably more challenging than losing weight. Most previous studies on weight loss maintenance have been conducted in Western countries and have assessed physical activity and sleep with the use of questionnaires. This preliminary study investigated the associations of objectively measured physical activity and sleep with weight loss maintenance among 25 Japanese adults who had intentionally lost ≥ 10% of their original weight. Participants wore tri-axial accelerometers on their waists and sleep monitors on their wrists for two weeks to measure their physical activity and sleep, respectively. A linear regression adjusted for sex, age, maximum weight, and time since losing weight was performed to investigate these associations. Participants had a mean body mass index of 23.4 kg/m^2^ and a median weight loss of 12.5%. Compared to those who maintained < 12.5% weight loss, those who maintained ≥ 12.5% weight loss slept longer (adjusted mean difference: 66.1 min/night, 95% confidence interval (CI): −14.0, 146.3 min/night, *p* = 0.10) and performed less moderate-to-vigorous physical activity (adjusted mean difference: −21.7 min/day, 95% CI: −58.0, 14.5 min/day, *p* = 0.22). Though statistical power was limited, sleep behavior may be an important modifiable factor that facilitates weight loss maintenance. Our findings can be used to establish a well-designed study to confirm this association.

## 1. Introduction

Despite well-established health benefits, weight loss maintenance is considerably more challenging than weight loss [1]. Our research team previously developed a group-based weight loss program in which weight loss was shown to confer cardiometabolic benefits such as reduced blood pressure and insulin resistance [2,3,4]. However, many individuals who succeed in losing weight do not maintain their weight loss in the long term and may regain the weight or gain even more weight than they had lost, thus losing the health benefits associated with weight loss [5]. In Japan, the prevalence of obesity, defined as a body mass index (BMI) ≥ 25 kg/m^2^, has been steadily increasing, particularly among middle-aged men; approximately 30% of Japanese men (age 40–69) are classified as obese [6]. Considering the high prevalence of obesity, the Japanese Ministry of Health, Labour and Welfare implemented a national health checkup and lifestyle intervention program in April 2008 that targeted individuals with metabolic syndrome [7], a condition characterized by abdominal obesity, hypertension, dyslipidemia, and hyperglycemia. In this context, maintaining weight loss is a public health priority for Japanese adults with obesity.

The US National Weight Control Registry (NWCR), which was established in 1993, is a representative observational study on the behavioral determinants of weight loss maintenance. The NWCR has included individuals who had maintained a weight loss of ≥ 13.6 kg (≥ 30 lb) for > 1 year [8,9]. A series of NWCR studies found that individuals who maintain weight loss in the long term engage in high levels of physical activity [10], consume low amounts of energy and fat [11], tend to eat breakfast [12], maintain a consistent dietary pattern [13], and weigh themselves frequently [14]. Individuals who do not maintain their weight loss are more likely to have a history of depression and binge-eating [15] and are more likely to have a medical disorder such as heart disease, hypertension, or diabetes prior to beginning weight loss [16].

Most previous studies, including those of the NWCR, have been conducted primarily among people of European origin and have used questionnaires to investigate associations between behavioral factors (including physical activity, sedentary behavior, and sleep behavior) and weight loss maintenance [10,17]. Because Japanese people tend to have different eating habits [18], physical activity environments [19], cultural backgrounds, and body sizes from people in Western countries, results from previous studies performed on Western populations are not generalizable to the Japanese population. Currently, the amount of information on the behavioral factors that are associated with maintaining weight loss among Japanese people is very limited. The aim of this preliminary study was to evaluate the associations between objectively measured physical activity, sedentary behaviors, sleeping behaviors, and weight loss maintenance among Japanese adults. The findings from this study will help to inform future studies with larger sample sizes.

## 2. Methods

### 2.1. Design, Settings, and Participants

We conducted a cross-sectional study in Tsukuba City in Ibaraki Prefecture from September to October 2017. Participants were recruited by advertising in a free local newspaper that invited interested participants to contact the research team by phone or e-mail. Following a brief screening over the phone or email, potential participants were invited for a 1 h introductory session where they provided written informed consent. To be eligible to participate, individuals were required to be between the ages of 18 and 65, and they were required to have achieved an intentional weight loss of ≥10% of their initial body weight, other than as a result of pregnancy or illness [20]. Potential participants were excluded if they were likely to have an irregular schedule, which included factors such as shift work, late-night work, or long business trips, during the study period. The study protocol was approved by the Institutional Review Board of the Graduate School of Arts and Sciences at The University of Tokyo (approval number: 556). This study was conducted in full accordance with the Declaration of Helsinki. Participants were compensated with 5000 JPY (approximately 46 USD in 2019) upon completion of the study.

### 2.2. Measurements

Participants were provided with a tri-axial accelerometer and a sleep monitor, and they were instructed to wear them simultaneously for a continuous two-week period. When they returned the accelerometer and sleep monitor, participants completed a self-administered questionnaire that provided information on their sociodemographic and lifestyle characteristics, current anthropometrics, weight history, weight control, and eating behaviors.

#### 2.2.1. Physical Activity and Sedentary Behavior

Physical activity and sedentary behavior were evaluated by using a validated tri-axial accelerometer (Active style Pro HJA-750C; Omron Healthcare, Kyoto, Japan) [21]. The participants were instructed to continuously wear the accelerometer on the left side of their waist, except while sleeping. For safety reasons, they were also asked to take off the device when engaging in water-based activities (e.g., bathing or swimming) or participating in contact sports (e.g., soccer or rugby). The accelerometer data were processed by a custom-written macro program specifically designed to compile data from the Active Style Pro accelerometers. The accelerometer estimated the intensity of physical activity (expressed as metabolic equivalents [METs]) in 60 s segments, and we used these data to determine the amount of time spent engaging in sedentary behavior (1.0–1.5 METs) and moderate-to-vigorous physical activity (MVPA, ≥ 3 METs) by using a validated algorithm [21]. A valid record was defined as ≥ 10 h/d of wear time. Once valid records had been collected for ≥ 7 days, various activity and sedentary measures were calculated. The computed variables included step counts, the amount of sedentary behavior, and MVPA. Sedentary behavior lasting ≥ 30 min and MVPA lasting ≥ 10 min was also calculated according to current international and domestic physical activity guidelines [22,23,24].

#### 2.2.2. Sleep

Participants were asked to continuously wear the sleep monitor (GT3X+; ActiGraph, LLC, Pensacola, FL, USA) on their non-dominant wrist, except while bathing or participating in other water-based activities [25]. The device was configured to collect data in 60 s segments at 30 Hz. Once the data had been collected, an investigator with expertise in actigraphy manually entered the bed/rise times by visual inspection. Each segment during a given sleep episode was scored as asleep or awake by using the Cole–Kripke algorithm [26], which had previously been validated in a sample of older adults. ActiLife version 6.13.3 software (Pensacola, FL, USA) was then used to generate the following sleep variables: total sleep time, sleep latency (minutes lying in bed before sleep onset), sleep efficiency (percentage of time asleep while in bed), wake after sleep onset (minutes awake between sleep onset and wake time), and the number of awakenings.

#### 2.2.3. Sociodemographic and Lifestyle Characteristics

Participants used a self-administered questionnaire to report their sex, age, employment status (full-time worker or other), educational attainment (college graduate or less), marital status (currently married or not), current smoking status (yes or no), and alcohol use (yes or no).

#### 2.2.4. Current Anthropometrics, Weight History, and Weight Control Behavior

Participants self-reported their current height and body weight, and their BMI was computed as weight (kg)/height^2^ (m^2^). They also reported their lifetime maximum body weight and their age at the time of reaching that weight. These data were subsequently used to calculate the number of years since reaching maximum body weight. Participants also answered questions on weight control behaviors such as the number of weight loss attempts and self-weighing frequency (several times/day, once/day, several times/week or less).

#### 2.2.5. Eating Behavior

Survey questions on eating behavior included the usual frequencies of eating meals, snacks, breakfast, fast foods, and eating out (all measured as times/week). Participants also completed Sakata’s Obesogenic Eating Behavior Scale questionnaire [27]. The scale is comprised of 30 items with 7 subscales (cognition of constitution, motivation for eating, substitute eating and drinking, feelings of satiety, eating style, meal contents, and eating rhythm abnormalities), and each item is measured on a 4-point Likert scale with response categories ranging from 1 (“no”) to 4 (“yes”). Higher scores indicate higher levels of obesogenic eating behavior.

### 2.3. Statistical Analyses

Since this was a preliminary study intended to inform a future survey, we did not calculate a priori statistical power. All data were processed and analyzed by using R version 3.2.4 for Windows, an open-source statistical software (R Foundation for statistical computing, Vienna, Austria). *P* values < 0.05 were considered statistically significant. We calculated the weight loss maintenance level as follows: (maximum weight—current weight)/maximum weight. A greater weight loss maintenance level indicates greater success at maintaining weight loss. Based on the median weight loss maintenance level, participants were divided into “successful maintainers” (≥ 12.5%) and “less successful maintainers” (< 12.5%). Participant characteristics are summarized as means and standard deviations for continuous variables, as well as frequencies and percentages for categorical variables. Unequal variance *t*-tests (Welch *t*-tests) were used to assess between-group differences, and χ^2^ tests or Fisher’s exact tests (to contingency tables with cell counts of < 5) were used to compare proportions. Associations between the degree of weight loss maintenance (i.e., successful maintainers vs. less successful maintainers [reference category]) and activity-, sedentary-, and sleep-related variables were investigated by using multiple linear regression analyses with and without adjustment for confounding variables. The regression models were adjusted for sex, age, lifetime maximum weight [28], and the number of years since the age of the maximum body weight [9].

## 3. Results

### 3.1. Participant Flow and Characteristics

We received 29 phone calls or emails from potential participants. After a brief remote screening for eligibility, 26 candidates were invited for a face-to-face introductory session. Of those who were invited, 25 attended the session and gave written informed consent. All 25 participants successfully completed the study and provided valid data, except for one participant whose sleep data were invalid due to a malfunction of their sleep monitor. The mean age (standard deviation) of the study participants was 48.6 (8.7) years old, and 64% were female.

Table 1 summarizes participant characteristics according to their weight loss maintenance level. Though not significant, successful maintainers were less likely to work full-time (*p* = 0.15) and had a lower weight (*p* = 0.07) and BMI (*p* = 0.10) compared to less successful maintainers. There were no other notable differences in sociodemographic or lifestyle characteristics between the two groups.

### 3.2. Physical Activity, Sedentary Behavior, and Sleep Behavior

Table 2 shows a comparisons of physical activity, sedentary behavior, and sleep behavior according to the weight loss maintenance category. Successful maintainers had a longer total sleep time (*p* = 0.07) and a shorter sleep latency (*p* = 0.22) than less successful maintainers. They also spent less time performing MVPA (*p* = 0.21), had fewer 10-min periods of MVPA (*p* = 0.23), and had lower step counts (*p* = 0.26).

Table 3 shows the associations between successful weight loss maintenance status and the level of physical activity, sedentary behavior, as well as sleeping behavior before and after adjustment for potential confounding variables. In the crude model, successful weight loss maintenance was associated with a longer total sleep time (*p* = 0.08). This association was slightly attenuated in the adjusted model (*p* = 0.10), but, on average, the successful maintainers slept for 66 min longer each night than the less successful maintainers.

## 4. Discussion

This cross-sectional preliminary study explored the associations of objectively measured levels of physical activity, sedentary behavior, and sleeping behavior with weight loss maintenance among Japanese adults. Though the analysis did not reveal any statistically significant associations, the results suggest that successful weight loss maintenance is associated with longer sleep times, lower levels of physical activity, and greater levels of sedentary behavior.

Previous studies have shown an association between longer sleep times and successful weight loss maintenance. Ross et al. [17] found that NWCR participants with longer sleep durations (subjectively quantified by a questionnaire) were more likely to be successful at maintaining their weight loss than those with shorter sleep durations. Another prospective cohort study of Japanese adults showed that participants with ≤5 h sleep per night were more likely to gain weight and to be obese than those who slept for longer [29]. In contrast to our study, these previous studies did not use an objective measure of sleep characteristics; instead, they measured sleep characteristics by using the Pittsburgh Sleep Quality Index [30] or a customized questionnaire. Though our study was preliminary in nature, the objective methods of measurements that we used reduced reporting bias and provided results that were consistent with those of previous studies.

Other studies have also found an association between short sleep durations and obesity. A systematic review [31] that summarized the findings of thirty-six studies found that short sleep durations are associated with higher body weights. St-Onge et al. [30] found that obesity and higher BMIs are more prevalent among those who sleep for a shorter amount of time (< 7 h/night) compared to those who sleep for an adequate amount of time (7–8 h/night). In our study, less successful weight maintainers had a lower total sleep time (approximately 6 h/night) than successful maintainers (approximately 7 h/night). 

The association between short sleep duration and obesity may be explained by several behavioral and physiological reasons. First, less sleep time means that individuals are awake for longer, thus allowing for more opportunities to experience hunger and eat. Shorter sleep durations have been associated with a higher dietary energy intake (300–550 kcal/d) [32], a high fat diet, and fast food consumption [33]. Furthermore, a lack of sleep causes an elevation in serum ghrelin and a reduction in serum leptin, both of which may influence peripheral regulators of hunger. The increased energy intake associated with short sleep duration may lead to difficulties with weight management. Though we did not measure appetite-related hormones such as leptin and ghrelin, it is possible that these mechanisms play a role in the association between weight maintenance and total sleeping time. Further research is warranted to better understand the association between sleeping behavior and weight loss maintenance, including the control of appetite-related hormones.

The association between physical activity and weight loss maintenance should be mentioned. A systematic review and meta-analysis [34] found that people who exercise are likely to sleep longer. Previous studies [10,35] have shown an association between increasing physical activity and weight loss maintenance. Contrary to the findings of other studies, the successful maintainers in our study spent less total time performing MVPA and had fewer bouts lasting ≥10 min than less successful maintainers. We are unable to explain this lack of association between level of physical activity and weight loss maintenance. One possible explanation may be that sleep duration is strongly associated with dietary energy intake. Catenacci et al. [36] reported that, among NWCR cohort members, those who increased both their physical activity and their energy intake were more likely to have regained the body weight that they had lost by the time of the three-year follow up. This suggests that energy intake has a greater effect on body weight than physical activity. Other studies [37,38] have suggested that women who engage in high intensity exercise may have a higher post-exercise energy intake, especially from fat calories. In our study, less successful maintainers had a higher total score of obesogenic eating behavior than successful maintainers; however, no significant associations between obesogenic eating behaviors and successful weight loss maintenance were found. Though we did not measure energy intake, these higher scores may have been due to a positive energy balance at high levels of energy intake. In future studies, the energy intake of successful and less successful weight loss maintainers should be measured.

Our study had several strengths. First, physical activity and sedentary behavior were measured with an activity monitor, and sleeping behavior was measured with a sleep monitor. In contrast, most previous studies have assessed these factors by only using questionnaires [10,17]. Furthermore, our measurement period (two weeks) for activity, sedentary, and sleeping behaviors was longer than that of previous studies (one week) [39,40]. Therefore, we believe that our measurement is more likely to reflect habitual physical activity as well as sedentary and sleeping behaviors.

This study also has some noteworthy limitations. First, the number of participants was much lower than in previous studies. Without an adequate number of participants, the statistical power to detect significant associations was limited. Caution should be used when interpreting the bivariate analysis results presented in Table 1 and Table 2, as some of the frequencies are below 5. Furthermore, because the adjusted analysis results presented in Table 3 might not fully control for confounding factors, there may be some residual confounders. Despite these limitations, our findings can be used to help estimate the required sample size for a larger future study. Based on the observed determination coefficient of 0.14 in the adjusted model (Table 3) with a significance level of 5% and a power of 80%, a total of at least 100 participants is required to detect a significant association. We enrolled individuals who had achieved an intentional weight loss of ≥10%. This restrictive weight loss definition might cause difficulty in recruiting a sufficient number of participants. The Japan Society for the Study of Obesity recommends a weight loss ≥5% for improving cardiometabolic risk factors for Japanese adults with obesity [41]. Adopting this ≥5% weight loss definition may make the recruitment process easier and contribute to the effective expansion of the sample size in future large-scale studies. The second limitation was the cross-sectional study design, which did not allow us to determine the directionality of associations. Thus, it is unclear whether behavioral factors including sleep, physical activity, and eating behavior affected weight loss maintenance, or whether weight loss maintenance affected those behaviors. Third, while there may be an association between sleeping habits and dietary energy intake, we did not measure energy intake. Previous studies [31,32,33] have shown a negative association between sleep duration and dietary energy intake. We cannot confirm or refute those findings. The next step is to introduce detailed measures of dietary energy intake (e.g., a weighed dietary record) for several days and to control for the confounding effect of dietary energy intake. Fourth, all participants in our study were able to maintain their weight loss. Therefore, we did not compare individuals who lost weight and then regained it. Future studies should include a substantial proportion of individuals who regained their weight to clarify the behavioral factors that are related to successful weight loss maintenance. Fifth, we did not obtain information about primary sleep disorders (e.g., obstructive sleep apnea syndrome, periodic limb movement, and insomnia) for participants. Finally, some measures, such as weight and height, maximum body weight, and time since attainment of maximum body weight, were obtained from self-reported measures and therefore were not validated. As a result, participants may have underreported their body weight status. Further longitudinal studies are needed to objectively measure body weight status.

## 5. Conclusions

Participants who were successful at maintaining their weight loss tended to sleep longer than those who were less successful; however, no significant associations were found. This finding is less likely to be biased because we used objective measures (physical activity, sedentary behavior, and sleep behavior) to assess the association. However, weight loss maintenance measurements were based on self-reporting. Our findings will be helpful to design a large cohort study to confirm this association.

## Figures and Tables

**Table 1:** Participant characteristics according to their weight loss maintenance category.

Characteristics	Overall (*n* = 25)	Weight Loss Maintenance		*p*-Value
		< 12.5% (*n* = 12)	≥ 12.5% (*n* = 13)	
**Sociodemographic and Lifestyle**				
Female sex, n (%)	16 (64.0)	6 (50.0)	10 (76.9)	0.23
Age, years	48.6 (8.7)	49.2 (10.7)	48.2 (6.8)	0.78
Full-time worker, n (%)	14 (56.0)	9 (75.0)	5 (38.5)	0.11
College graduate, n (%)	11 (44.0)	6 (50.0)	5 (38.5)	0.70
Current marriage, n (%)	21 (84.0)	11 (91.7)	10 (76.9)	0.65
Current smoking, n (%)	2 (8.0)	1 (8.3)	1 (7.7)	1.00
Current alcohol drinking, n (%)	12 (48.0)	6 (50.0)	6 (46.2)	1.00
**Anthropometrics**				
Height, cm	163.2 (8.0)	164.8 (8.7)	161.6 (7.2)	0.33
Weight, kg	62.6 (11.1)	66.9 (11.8)	58.7 (9.1)	0.07
Body mass index, kg/m^2^	23.4 (3.1)	24.5 (2.8)	22.5 (3.1)	0.10
Body mass index ≥25 kg/m^2^, n (%)	8 (32.0)	5 (41.7)	3 (23.1)	0.41
**Weight History**				
Maximum lifetime weight, kg	72.3 (13.4)	72.2 (13.3)	72.3 (14.0)	0.98
Age at maximum weight, years	37.8 (10.9)	38.4 (12.8)	37.2 (9.3)	0.80
Years since age at maximum weight, years	10.8 (10.3)	10.8 (11.0)	10.9 (10.1)	0.97
**Weight Control Behavior**				
Weight loss attempts, times	3.20 (2.83)	3.75 (3.86)	2.69 (1.32)	0.38
Self-weighing frequency, n (%)				1.00
Several times/day	4 (16.0)	2 (16.7)	2 (15.4)	
Once/day	11 (44.0)	5 (41.7)	6 (46.2)	
Several times/week or less	10 (40.0)	5 (41.7)	5 (38.5)	
**Eating Behavior**				
Eating meal (3 times/day), n (%)	22 (88.0)	10 (83.3)	12 (92.3)	0.94
Eating snacks (none), n (%)	9 (36.0)	4 (33.3)	5 (38.5)	1.00
Eating breakfast, times/week	5.86 (2.02)	5.54 (2.33)	6.15 (1.72)	0.47
Eating out, times/week	1.46 (1.83)	1.29 (1.29)	1.62 (2.26)	0.66
Eating fast foods (none), times/week, n (%)	19 (76.0)	9 (75.0)	10 (76.9)	1.00
**Obesogenic Eating Behavior Score, points**				
Cognition of constitution (3–12 points)	8.20 (2.38)	8.42 (2.57)	8.00 (2.27)	0.67
Motivation for eating (3–12 points)	7.92 (2.47)	8.08 (2.15)	7.77 (2.80)	0.76
Substitute eating and drinking (6–24 points)	16.6 (3.9)	17.6 (3.9)	15.6 (3.7)	0.21
Feelings of satiety (5–20 points)	14.6 (3.5)	14.8 (3.2)	14.4 (3.8)	0.80
Eating style (3–12 points)	7.04 (1.43)	6.75 (1.14)	7.31 (1.65)	0.33
Meal contents (5–20 points)	10.4 (2.9)	11.2 (2.5)	9.7 (3.2)	0.21
Eating rhythm abnormalities (5–20 points)	9.36 (3.20)	9.33 (3.42)	9.38 (3.12)	0.97
Total score (30–120 points)	74.0 (14.0)	76.1 (13.4)	72.2 (14.9)	0.49

Data are shown as mean (standard deviation) for continuous variables or frequency (percentage) for categorical variables. The <12.5% category corresponds to “less successful,” and the ≥12.5% category corresponds to “successful.”

**Table 2:** Comparison of levels of physical activity, sedentary behavior, and sleeping behavior, according to the weight loss maintenance category.

Characteristics	Overall (*n* = 25)	Weight Loss Maintenance		*p*-Value
		< 12.5% (*n* = 12)	≥ 12.5% (*n* = 13)	
**Physical Activity and Sedentary Behavior**				
Valid days, day	12.1 (1.1)	12.4 (0.7)	11.8 (1.3)	0.24
Wear time, min/day	933.6 (103.3)	944.3 (90.7)	924.6 (115.9)	0.64
Sedentary behavior, min/day	489.0 (117.4)	470.4 (132.4)	504.8 (105.9)	0.50
Sedentary behavior lasting ≥30 min, min/day	164.2 (80.6)	151.5 (80.4)	174.8 (82.5)	0.49
MVPA, min/day	79.0 (38.5)	90.6 (49.9)	69.2 (23.0)	0.21
MVPA lasting ≥10 min, min/day	21.5 (19.5)	27.1 (25.2)	16.7 (12.1)	0.23
Step counts, step/day	9019 (2549)	9656 (2176)	8480 (2797)	0.26
**Sleeping Behavior**				
Sleep episode, night	13.8 (3.2)	14.2 (2.8)	13.5 (3.6)	0.63
Total sleep time, min/night	390.9 (84.9)	357.6 (63.9)	419.1 (92.4)	0.07
Sleep latency, min/night	6.40 (1.90)	6.93 (1.97)	5.95 (1.79)	0.22
Sleep efficiency, %	88.0 (3.8)	87.3 (3.4)	88.6 (4.1)	0.41
Wake after sleep onset, min/night	46.8 (18.0)	44.6 (15.2)	48.7 (20.4)	0.59
Number of awakenings, n/night	17.6 (6.0)	17.0 (5.8)	18.2 (6.4)	0.65

Data are shown as mean (standard deviation). The sleep data were available for 24 participants (12 in each group). The <12.5% category corresponds to “less successful,” and the ≥12.5% category corresponds to “successful.” Abbreviation: MVPA, moderate-to-vigorous physical activity.

**Table 3:** Associations between successful weight loss maintenance and levels of physical activity, sedentary behavior, and sleeping behavior (*n* = 25).

Dependent Variables	Crude			Adjusted		
	β	95% CI	*p*-Value	β	95% CI	*p*-Value
**Sedentary Behavior**						
Sedentary behavior, min/day	30.0	(−66.5, 126.5)	0.53	61.3	(−41.5, 164.1)	0.23
Sedentary behavior lasting ≥ 30 min, min/day	28.8	(−38.2, 95.7)	0.38	38.7	(−37.9, 115.2)	0.30
**Physical Activity**						
MVPA, min/day	−16.3	(−48.4, 15.9)	0.31	−21.7	(−58.0, 14.5)	0.22
MVPA lasting ≥ 10 min, min/day	−8.1	(−24.3, 8.1)	0.31	−7.9	(−26.6, 10.8)	0.39
Step counts, step/day	−784	(−2979, 1410)	0.47	−727	(−3245, 1791)	0.55
**Sleeping Behavior**						
Total sleep time, min/night	61.5	(−7.1, 130.1)	0.08	66.1	(−14.0, 146.3)	0.10
Sleep latency, min/night	−0.98	(−2.57, 0.61)	0.21	−0.43	(−2.11, 1.25)	0.60
Sleep efficiency, %	1.29	(−1.96, 4.53)	0.42	0.92	(−2.70, 4.55)	0.60
Wake after sleep onset, min/night	4.0	(−11.5, 19.5)	0.59	6.4	(−11.0, 23.8)	0.45
Number of awakenings, n/night	1.14	(−4.07, 6.35)	0.65	2.24	(−2.91, 7.39)	0.37

The sleep data were available for 24 participants (12 in each group). The regression coefficients for each dependent variable compare those who maintained a weight loss of ≥12.5% with those who maintained a weight loss of <12.5% as the reference category. The model was adjusted for sex, age, age at lifetime maximum weight, lifetime maximum weight, and years since lifetime maximum weight. Abbreviations: β, regression coefficient; CI, confidence interval.

## References

[1] Kraschnewski J.L., Boan J., Esposito J., Sherwood N.E., Lehman E.B., Kephart D.K., Sciamanna C.N. (2010). Long-Term Weight Loss Maintenance in the United States. Int. J. Obes. (Lond.).

[2] Sasai H., Katayama Y., Nakata Y., Ohkubo H., Tanaka K. (2009). Obesity Phenotype and Intra-Abdominal Fat Responses to Regular Aerobic Exercise. Diabetes Res. Clin. Pract..

[3] Sasai H., Katayama Y., Nakata Y., Eto M., Tsujimoto T., Ohkubo H., Tanaka K. (2010). The Effects of Vigorous Physical Activity on Intra-Abdominal Fat Levels: A Preliminary Study of Middle-Aged Japanese Men. Diabetes Res. Clin. Pract..

[4] Nakata Y., Sasai H. (2015). Current Review of Intervention Studies on Obesity and the Role of Physical Activity in Weight Control. J. Phys. Fit. Sports Med..

[5] Nakata Y., Okada M., Hashimoto K., Harada Y., Sone H., Tanaka K. (2014). Weight Loss Maintenance for 2 Years After a 6-Month Randomised Controlled Trial Comparing Education-Only and Group-Based Support in Japanese Adults. Obes. Facts.

[6] Ministry of Health, Labour and Welfare National Health and Nutrition Survey. http://www.mhlw.go.jp/stf/houdou/0000177189.html.

[7] Ministry of Health, Labour and Welfare Specific Health Checkups and Specific Health Guidance. http://www.mhlw.go.jp/english/wp/wp-hw3/dl/2-007.pdf.

[8] Klem M.L., Wing R.R., McGuire M.T., Seagle H.M., Hill J.O. (1997). A Descriptive Study of Individuals Successful at Long-Term Maintenance of Substantial Weight Loss. Am. J. Clin. Nutr..

[9] Thomas J.G., Bond D.S., Phelan S., Hill J.O., Wing R.R. (2014). Weight-Loss Maintenance for 10 Years in the National Weight Control Registry. Am. J. Prev. Med..

[10] Catenacci V.A., Ogden L.G., Stuht J., Phelan S., Wing R.R., Hill J.O., Wyatt H.R. (2008). Physical Activity Patterns in the National Weight Control Registry. Obesity (Silver Spring).

[11] Shick S.M., Wing R.R., Klem M.L., McGuire M.T., Hill J.O., Seagle H. (1998). Persons Successful at Long-Term Weight Loss and Maintenance Continue to Consume a Low-Energy, Low-Fat Diet. J. Am. Diet. Assoc..

[12] Wyatt H.R., Grunwald G.K., Mosca C.L., Klem M.L., Wing R.R., Hill J.O. (2002). Long-Term Weight Loss and Breakfast in Subjects in the National Weight Control Registry. Obes. Res..

[13] Gorin A.A., Phelan S., Wing R.R., Hill J.O. (2004). Promoting Long-Term Weight Control: Does Dieting Consistency Matter?. Int. J. Obes. Relat. Metab. Disord..

[14] Butryn M.L., Phelan S., Hill J.O., Wing R.R. (2007). Consistent Self-Monitoring of Weight: A Key Component of Successful Weight Loss Maintenance. Obesity (Silver Spring).

[15] Klem M.L., Wing R.R., McGuire M.T., Seagle H.M., Hill J.O. (1998). Psychological Symptoms in Individuals Successful at Long-Term Maintenance of Weight Loss. Health Psychol..

[16] McGuire M.T., Wing R.R., Klem M.L., Seagle H.M., Hill J.O. (1998). Long-Term Maintenance of Weight Loss: Do People Who Lose Weight Through Various Weight Loss Methods Use Different Behaviors to Maintain Their Weight?. Int. J. Obes. Relat. Metab. Disord..

[17] Ross K.M., Graham Thomas J.G., Wing R.R. (2016). Successful Weight Loss Maintenance Associated with Morning Chronotype and Better Sleep Quality. J. Behav. Med..

[18] Zhou B.F., Stamler J., Dennis B., Moag-Stahlberg A., Okuda N., Robertson C., Zhao L., Chan Q., Elliott P., Intermap Research Group (2003). Nutrient intakes of middle-aged men and women in China, Japan, United Kingdom, and United States in the late 1990s: The INTERMAP study. J. Hum. Hypertens..

[19] Sallis J.F. (2011). Environmental and Policy Research on Physical Activity is Going Global. Res. Exerc. Epidemiol..

[20] Wing R.R., Hill J.O. (2001). Successful Weight Loss Maintenance. Annu. Rev. Nutr..

[21] Ohkawara K., Oshima Y., Hikihara Y., Ishikawa-Takata K., Tabata I., Tanaka S. (2011). Real-Time Estimation of Daily Physical Activity Intensity by a Triaxial Accelerometer and a Gravity-Removal Classification Algorithm. Br. J. Nutr..

[22] Kurita S., Yano S., Ishii K., Shibata A., Sasai H., Nakata Y., Fukushima N., Inoue S., Tanaka S., Sugiyama T. (2017). Comparability of Activity Monitors Used in Asian and Western-Country Studies for Assessing Free-Living Sedentary Behaviour. PLoS ONE.

[23] Troiano R.P., Berrigan D., Dodd K.W., Mâsse L.C., Tilert T., McDowell M. (2008). Physical Activity in the United States Measured by Accelerometer. Med. Sci. Sports Exerc..

[24] World Health Organization Global Recommendations on Physical Activity for Health. https://www.who.int/dietphysicalactivity/factsheet_recommendations/en/.

[25] Kim M., Sasai H., Kojima N., Kim H. (2015). Objectively Measured Night-To-Night Sleep Variations Are Associated with Body Composition in Very Elderly Women. J. Sleep Res..

[26] Cole R.J., Kripke D.F., Gruen W., Mullaney D.J., Gillin J.C. (1992). Automatic Sleep/Wake Identification from Wrist Activity. Sleep.

[27] Sakata T. (1996). Treatment Manual for Morbid Obesity.

[28] Kobayashi D., Takahashi O., Deshpande G.A., Shimbo T., Fukui T. (2012). Association Between Weight Gain, Obesity, and Sleep Duration: A Large-Scale 3-Year Cohort Study. Sleep Breath..

[29] Ogden L.G., Stroebele N., Wyatt H.R., Catenacci V.A., Peters J.C., Stuht J., Wing R.R., Hill J.O. (2012). Cluster Analysis of the National Weight Control Registry to Identify Distinct Subgroups Maintaining Successful Weight Loss. Obesity (Silver Spring).

[30] Buysse D.J., Reynolds III C.F., Monk T.H., Berman S.R., Kupfer D.J. (1989). The Pittsburgh Sleep Quality Index: A New Instrument for Psychiatric Practice and Research. Psychiatry Res..

[31] Patel S.R., Hu F.B. (2008). Short Sleep Duration and Weight Gain: A Systematic Review. Obesity (Silver Spring).

[32] St-Onge M.P. (2017). Sleep—Obesity Relation: Underlying Mechanisms and Consequences for Treatment. Obes. Rev..

[33] St-Onge M.P. (2013). The Role of Sleep Duration in the Regulation of Energy Balance: Effects on Energy Intakes and Expenditure. J. Clin. Sleep Med..

[34] Lang C., Kalak N., Brand S., Holsboer-Trachsler E., Pühse U., Gerber M. (2016). The Relationship Between Physical Activity and Sleep from Mid Adolescence to Early Adulthood. A Systematic Review of Methodological Approaches and Meta-Analysis. Sleep Med. Rev..

[35] Swift D.L., Johannsen N.M., Lavie C.J., Earnest C.P., Church T.S. (2014). The Role of Exercise and Physical Activity in Weight Loss and Maintenance. Prog. Cardiovasc. Dis..

[36] Catenacci V.A., Odgen L., Phelan S., Thomas J.G., Hill J., Wing R.R., Wyatt H. (2014). Dietary Habits and Weight Maintenance Success in High Versus Low Exercisers in the National Weight Control Registry. J. Phys. Act. Health.

[37] Blundell J.E., Gibbons C., Caudwell P., Finlayson G., Hopkins M. (2015). Appetite Control and Energy Balance: Impact of Exercise. Obes. Rev..

[38] George V.A., Morganstein A. (2003). Effect of Moderate Intensity Exercise on Acute Energy Intake in Normal and Overweight Females. Appetite.

[39] Ostendorf D.M., Lyden K., Pan Z., Wyatt H.R., Hill J.O., Melanson E.L., Catenacci V.A. (2018). Objectively Measured Physical Activity and Sedentary Behavior in Successful Weight Loss Maintainers. Obesity.

[40] Catenacci V.A., Grunwald G.K., Ingebrigtsen J.P., Jakicic J.M., McDermott M.D., Phelan S., Wing R.R., Hill J.O., Wyatt H.R. (2011). Physical Activity Patterns Using Accelerometry in the National Weight Control Registry. Obesity (Silver Spring).

[41] Japan Society for the Study of Obesity, Japan Society for the Study of Obesity (2016). Guidelines for the Management of Obesity Disease 2016.

